# Associations of VEGF-A-Related Variants with Adolescent Cardiometabolic and Dietary Parameters

**DOI:** 10.3390/nu15081884

**Published:** 2023-04-13

**Authors:** Maria Kafyra, Ioanna Panagiota Kalafati, Ioanna Gavra, Sophie Siest, George V. Dedoussis

**Affiliations:** 1 Department of Nutrition and Dietetics, School of Health Science and Education, Harokopio University, 17671 Athens, Greece; 2 Department of Nutrition and Dietetics, School of Physical Education, Sport Science and Dietetics, University of Thessaly, 42132 Trikala, Greece; 3 Interactions Gène-Environnement en Physiopathologie Cardio-Vasculaire (IGE-PCV), Université de Lorraine, 54000 Nancy, France; 4 Santorini Conferences (SCs) Association—For Research Innovation in Health, 54470 Bernecourt, France

**Keywords:** vascular endothelial growth factor A (VEGF-A), cardiometabolic profile, genetic risk score, adolescents, dietary patterns, genetic risk score

## Abstract

Previous research has allowed the identification of variants related to the vascular endothelial growth factor-A (VEGF-A) and their association with anthropometric, lipidemic and glycemic indices. The present study examined potential relations between key VEGF-A-related single-nucleotide polymorphisms (SNPs), cardiometabolic parameters and dietary habits in an adolescent cohort. Cross-sectional analyses were conducted using baseline data from 766 participants of the Greek TEENAGE study. Eleven VEGF-A-related SNPs were examined for associations with cardiometabolic indices through multivariate linear regressions after adjusting for confounding factors. A 9-SNP unweighted genetic risk score (uGRS) for increased VEGF-A levels was constructed to examine associations and the effect of its interactions with previously extracted dietary patterns for the cohort. Two variants (rs4416670, rs7043199) displayed significant associations (*p*-values < 0.005) with the logarithms of systolic and diastolic blood pressure (logSBP and logDBP). The uGRS was significantly associated with higher values of the logarithm of Body Mass Index (logBMI) and logSBP (*p*-values < 0.05). Interactions between the uGRS and specific dietary patterns were related to higher logDBP and logGlucose (*p*-values < 0.01). The present analyses constitute the first-ever attempt to investigate the influence of VEGF-A-related variants on teenage cardiometabolic determinants, unveiling several associations and the modifying effect of diet.

## 1. Introduction

Vascular endothelial growth factor A (VEGF-A) is involved in various biological functions, primarily as a major contributor to angiogenesis induction which extends its activities to cell proliferation, migration and even differentiation [1,2,3]. Due to its versatile roles in endothelial function [4], its involvement in activating the cortisol–adrenocorticotrophic hormone (ACTH) stress axis, its promotion of aldosterone [5] production as well as its multifactorial influence on energy homeostasis [2,6,7], insulin resistance [2,8] and cardiac function [9], VEGF-A is involved in various reciprocal relationships influencing cardiovascular and cardiometabolic risk factors such as glucose sensitivity, lipidemic profile, obesity and blood pressure.

Altered VEGF-A expression is observed in the presence of disturbed cardiometabolic states, denoting a requited relationship between the biomarker’s levels and disrupted cardiometabolic profile. For example, VEGF-A is known to be involved in glucose homeostasis, where both its over- and under-expression can affect glucose tolerance [8], as well as lipid metabolism, through its regulation of lipases and the creation of chylomicrons [7]. In a similar manner, VEGF-A is highly expressed in the adipose tissue, where an increase in the number of adipocytes signifies increased VEGF-A and subsequent angiogenesis and further cell proliferation and differentiation [1].

Circulating VEGF-A levels have been conclusively demonstrated as greatly heritable [10]. The past decades have marked the conduct of large meta-analyses of multiple genome-wide association studies (GWAS), revealing key variants significantly associated with the marker’s levels. More specifically, Debette and Visvikis-Siest et al. brought four key single-nucleotide polymorphisms (SNPs) to light, collectively explaining 48.7% of VEGF-A variation [10]. Subsequent studies have unveiled additional VEGF-A-related SNPs, which have, in turn, been further associated with adult cardiometabolic indices [11,12] and even the presence of neurodegenerative disorders such as Alzheimer’s disease [13]. Selected VEGF-A-associated SNPs have even been directly linked to the presence of hypercholesterolemia and metabolic syndrome in adults [14,15]. In addition, the interplay between VEGF-A SNPs and dietary components has also been associated with multiple metabolic syndrome determinants [16,17]. An example of the importance of the interplay between VEGF-A, anthropometric indices and dietary compounds was recently highlighted in the finding that the effect of VEGF- A variants on circulating iron levels might depend on anthropometric indices [(i.e., Body Mass Index (BMI)] [18].

The present study constitutes the continuation of our team’s previous research aiming at exploring the effect of the interplay between genetic makeup and lifestyle habits on adolescent anthropometric, lipidemic and glycemic indices. In this context, the present findings concern the first-ever attempt to investigate the role of key VEGF-A-related variants exclusively on the cardiometabolic profile of adolescents, using data from the Greek TEENAGE Study. We hereby present the results of the analyses on selected target variants, the subsequent examinations of their cumulative effect in the form of an unweighted genetic risk score (uGRS) and its respective interactions with previously extracted dietary patterns on the teenagers’ cardiometabolic indices.

## 2. Materials and Methods

### 2.1. The TEENAGE Study

The present analyses constitute the next step in the research of our team’s Gutenberg Chair 2018 project, where building on our previous findings [19], we hereby present the subsequent examinations between genetic makeup and teenage cardiometabolic profile in the TEENAGE Study. The latter (TEENs of Attica: Genes and Environment) refers to the cross-sectional collection of various data from adolescent students conducted during the years 2008–2010 in Attica, Greece. The project was approved by the Institutional Review Board of Harokopio University of Athens, as well as the Greek Ministry of Education and Religious Affairs. All nodes conducted within the study took place adhering to the guidelines of the Declaration of Helsinki.

Details of the study protocol and characteristics have been previously extensively described elsewhere [20,21,22]. The TEENAGE desired target population were children and adolescents of 13–15 years of age attending the primary three classes of public secondary schools in the Attica region, coming from all groups and backgrounds [22]. Schools and participants were invited to be involved in the study from the pool of the teenage population of the GENDAI study [23]. The latter constituted a previous study also conducted and approved by Harokopio University of Athens, including children attending fifth and sixth grade of 1440 schools from a wide range of neighborhoods of different socioeconomic status across the Attica region [23]. Overall, 857 out of 1440 teenagers attending the participating schools were recruited for the purposes of the TEENAGE study [20,21]. The volunteers were recruited to the study after undergoing a briefing session on the study aims, their voluntary inclusion and the confidentiality measures surrounding their data [20,21]. Verbal consent by all adolescent participants and their respective guardians’ written consent was collected prior to study enrollment.

After enrollment in the study, all children and adolescents participated in a baseline, in-person session with healthcare professionals, where anthropometric, dietary, biochemical and lifestyle data were collected. Measurements of body and height were conducted for each individual in a barefoot state and with light clothes on, and the BMI was calculated as weight (kg)/height^2^ (m^2^). Waist circumference was measured in centimeters using a non-extensible soft tape, and body fat was evaluated by measuring the triceps and subscapular skinfolds. Dietary intake was assessed via conduct of a 24 h recall for the day prior to recruitment and the completion of a questionnaire for meal patterns and eating behavior. A second recall was conducted via telephone in the 10 days after the baseline session. Physical activity habits were assessed via the completion of a relative checklist for two non-consecutive days [20,21,22].

Moreover, DNA samples were collected for each participant and were further genotyped via the use of the Illumina HumanOmniExpress BeadChips (Illumina, San Diego, CA, USA) at the Wellcome Trust Sanger Institute, Hinxton, UK [20]. The imputation of the genotyped data was conducted using the Haplotype Reference Consortium (HRC) panel [20,24].

For the purposes of the present study, we used anthropometric, biochemical and genetic data from an initial pool of 766 participants with available data. We investigated associations between 11 VEGF-A-associated SNPs and various cardiometabolic indices. Pulse pressure (PP) was calculated to allow for comparisons with the previous findings, based on the available data for systolic and diastolic blood pressure (SBP and DBP, respectively) and via using the following formula:

$$\begin{array}{c} Pulse\;Pressure\;\left(PP\right)\\ =Systolic\;Blood\;Pressure\;\left(SBP,mmHg\right)\\ -Diastolic\;Blood\;Pressure\;\left(DBP,mmHg\right) \end{array}$$

Furthermore, we proceeded to construct an unweighted genetic risk score (uGRS) for VEGF-A using the target SNPs identified by Choi et al. For the purposes of the present analyses, we used the SNPs with the available data in the TEENAGE cohort (i.e., 9 out of 10 variants). The uGRS was constructed by scoring the risk alleles positively associated with the VEGF-A levels. We subsequently examined its respective relations with the cardiometabolic indices and further split the uGRS into two groups of high and low genetic risk for higher levels of VEGF based on the sample median value. Additionally, we proceeded to investigate the potential effect of interactions between the uGRS and the previously identified dietary patterns for the TEENAGE cohort [19] on the various indices.

### 2.2. Statistical Analyses

In the present analyses, we set out to investigate the potential impact of 11 VEGF-A-related target SNPs on cardiometabolic indices using available data from the Greek TEENAGE study (Table 1). Based on our team’s previously published findings [10,11], we chose to examine the rs4416670, rs6921438, rs10738760 and rs6993770 variants, which have been shown to collectively explain 48.7% of VEGF-A variability and have been further associated with multiple cardiometabolic indices in healthy populations [10]. We additionally included 7 more SNPs identified by Choe et al. as strongly associated with circulating VEGF-A levels, with available data in the TEENAGE cohort [11].

We used a threshold of 0.7 for the imputation INFO score for all SNPs included in the analyses. Quality control for sample and SNP exclusion criteria consisted of: (i) sample call rate at 95%; (ii) Hardy–Weinberg Equilibrium (HWE) exact *p* < 0.0001; and (iii) genotyping call rate at 99%. Before testing for associations, an assessment of the cardiometabolic variables’ distribution was carried out via the use of the Shapiro–Wilk and Kolmogorov–Smirnov tests. All variables not presenting a normal distribution were log-transformed. Hypothesis testing between cohort subgroups took place using the Mann–Whitney test. We investigated potential relations between the 11 target SNPs and the cardiometabolic parameters using linear regression analyses. Associations were examined after adjusting for 3 different models of confounding factors, namely: (i) Model 1, which consisted of adjustment for age and sex; (ii) Model 2, which further included exercise level; and (iii) Model 3, additionally incorporating the adjustment for the five previously extracted dietary patterns [19]. Multiple linear regression results for each SNP are presented as betas [regression coefficients (β)] and *p*-values. The threshold for statistical significance was set at 0.05. The adjusted threshold for multiple testing was set at 0.005 (0.05/11 components examined).

Following the associations explored for each SNP separately, we further used multiple linear regressions to examine the associations between the uGRS and the metabolic indices, as well as the potential effect of the interactions between the uGRS and the formerly extracted dietary patterns. Multiple linear regression results are presented as estimates [beta coefficients (β)] and standard error (SE). In the case of examining the interactions, the adjusted threshold for statistical significance was set at 0.01 (i.e., 0.05/5 components examined). All phenotypic analyses were conducted using the R Statistical Package [25], and genetic analyses were carried out with the Plink whole-genome association analysis toolset, version 1.9 [26].

## 3. Results

### 3.1. Population Characteristics

The characteristics of the population used have been previously described elsewhere [19]. This overall healthy population of 349 boys and 417 girls presented a median age of 13.30 years old (Table 2). The girls displayed an overall better cardiometabolic profile compared to boys, with the latter showing statistically significantly higher levels of SBP, PP, glucose and C-reactive protein (CRP) (*p*-value < 0.001). Additionally, girls demonstrated statistically significantly higher levels of high-density cholesterol (HDL) (*p*-value < 0.001). BMI, triglycerides, total cholesterol, SBP, while low-density cholesterol did not display any statistically significant differences between the two groups.

### 3.2. Associations between the 11 VEGF-A-Related SNPs and the Cardiometabolic Indices

Cross-sectional associations between the 11 SNPs and the various indices were assessed in participants with available data. Table 3 shows the multivariate linear regressions conducted for each of the 11 SNPs after adjustment for age and sex (Model 1), age, sex and exercise (Model 2) and age, sex, exercise and dietary pattern (Model 3). Our analyses showed statistically significant associations for two out of the eleven examined SNPs, namely the rs7043199 and the rs4416670 variants, with the latter having been found to explain 1,5% of the variance of VEGF-A levels in adults [7]. More specifically, the presence of the C allele of the latter was related, with a lower log of systolic blood pressure (logSBP) across all models (Model 1: β = −0.007, *p*-value = 0.002, Model 2: β = −0.007, *p*-value = 0.002, Model 3: β = −0.07, *p*-value = 0.0035). Another statistically significant but positive relation for logSBP was demonstrated for the A allele of the rs7043199 variant after adjusting for Model 2 (Model 2: β = 0.009, *p*-value = 0.004). The same SNP also displayed a statistically significant and positive association with log diastolic blood pressure (logDBP) after adjustment for Model 3 (Model 3: β = 0.0138, *p*-value = 0.0046).

### 3.3. Associations between the 9-SNP uGRS and the Cardiometabolic Indices

**Figure 1:**
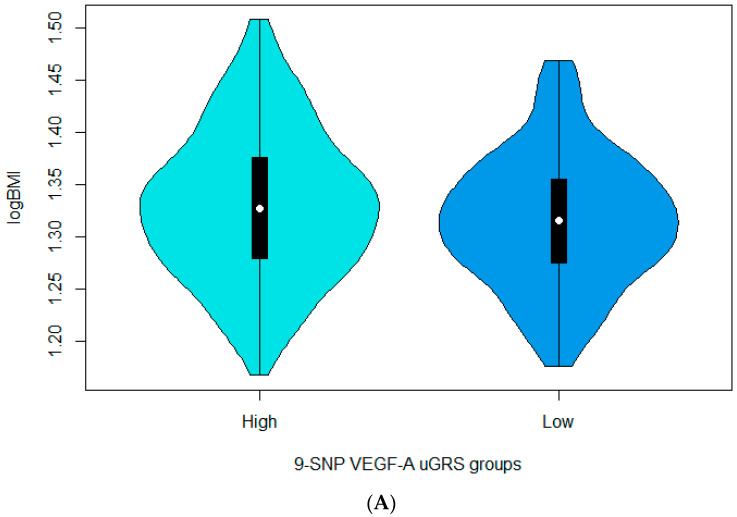

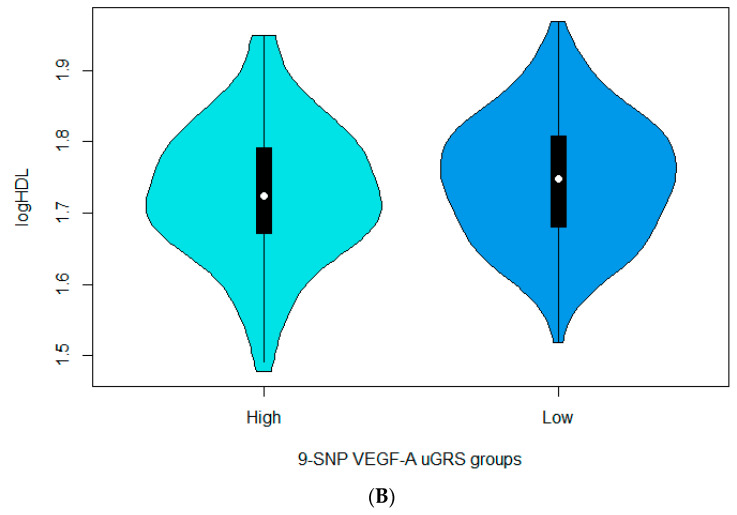

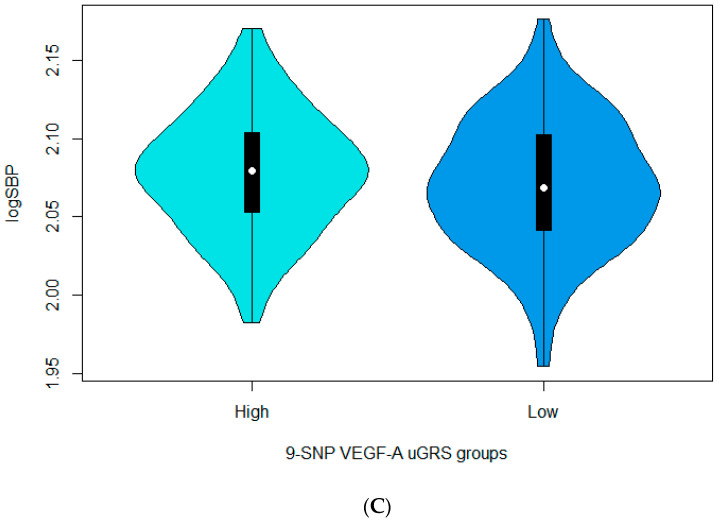
Violin plots depicting the distribution of (**A**) logBMI, (**B)** logSBP and (**C**) logHDL between the two groups of the 9-SNP VEGF-A unweighted GRS (low versus high), separated by the sample median (*p*-values < 0.05).

In the effort to examine the potential effect of uGRS in the formation of the investigated indices, we separated the 9-SNP uGRS into the two categories of “low” and “high” risk based on the sample median, where logBMI displayed statistically significant differences between the two groups (Figure 1), with individuals in the higher category presenting greater logBMI (*p*-value < 0.05), indicating that higher risk for increased VEGF-A levels is also associated with elevated logBMI. People in the higher percentile of uGRS also presented statistically significantly higher values of logSBP compared to the ones in the lower group (*p*-value < 0.05), also denoting that elevated risk for increased VEGF-A levels is further associated with increased logSBP. To boot, individuals with higher versus lower uGRS did display statistically significantly lower levels of logHDL (*p*-value < 0.05), highlighting an inverse association between increased risk for VEGF-A and levels of logHDL.

Furthermore, the creation of the 9-SNP uGRS was followed by association testing for all cardiometabolic indices explored via linear regressions after adjusting for age and sex (Model 1), age, sex and exercise (Model 2) and age, sex, exercise and dietary patterns (Model 3). Similar to the results deriving from the within-group comparisons and as shown in Table 4, significant associations were observed between higher uGRS values and increased levels of logBMI across all models (Model 1: β = 0.0044, *p*-value = 0.003, Model 2: β = 0.0043, *p*-value = 0.005, Model 3: β = 0.004, *p*-value = 0.009). Additionally, a statistically significant, positive association was also observed between the uGRS and logSBP, again after adjusting for all models (Model 1: β = 0.002, *p*-value = 0.03, Model 2: β = 0.019, *p*-value = 0.047, Model 3: β = 0.002, *p*-value = 0.037). The score was further negatively associated with logHDL levels after adjustment for age and sex (Model 1: β = −0.005, *p*-value = 0.032), an association which was not maintained after correcting for the additional confounders (exercise and dietary patterns).

### 3.4. Interactions between the uGRS and Dietary Patterns

After calculating the 9-SNP uGRS, we carried on to examine the potential associations between the cardiometabolic indices and their interactions with the five previously extracted patterns of food choices in the teenagers, namely the “Western Breakfast”, the “Legumes and Good Fat”, the “Homemade Meal”, the “Chickens and Sugars”, and the “Eggs and Fibers” patterns [19]. Table 5 shows the multivariate linear regressions carried out for each examined index and the interaction between the uGRS and each of the dietary patterns after adjusting for age, sex, uGRS and each dietary pattern (Model 1) and age, sex, and exercise. uGRS and each dietary pattern (Model 2).

As shown in the table, after evaluation based on the adjusted threshold (*p* = 0.01), the interaction between the uGRS and the “Western Breakfast” was associated with higher levels of logDBP (Model 1: β = 0.0060, *p*-value = 4.28 × 10^−5^, Model 2: β = 0.00568, *p*-value = 0.000239), suggesting that increased risk for high VEGF-A and adherence to a western-diet-like pattern is associated with elevated logDBP. A different nominally statistically significant, positive association was found for the interaction between the uGRS and consumption of the “Eggs and Fibers” pattern and increased levels of logGlucose after adjusting for age, sex, and exercise (Model 2: β = 0.00883, *p*-value = 0.0132), potentially indicating that elevated risk for increased VEGF-A and increased consumption of fiber-rich foods or eggs is associated with increased levels of logGlucose.

## 4. Discussion

The present study sought to conduct the first-ever attempt to investigate the role of VEGF-A-related variants on adolescent cardiometabolic profile, as well as their potential interplay with dietary habits. In this population of Greek teenagers, two VEGF-A-related SNPs, namely the rs7043199 and the rs4416670 variants, presented significant relations with blood pressure indices. Moreover, the 9-SNP uGRS constructed out of risk variants for higher VEGF-A levels was associated with higher levels of logBMI and logSBP but lower levels of logHDL. Furthermore, the exploration of associations between the uGRS and the teenagers’ dietary patterns revealed a significant relationship between the adherence to the “Western Breakfast” pattern and higher logDBP, as well as a nominal association for the “Eggs and Fibers” pattern and higher logGlucose.

In our sample, the negatively associated with VEGF-A levels C allele of the rs4416670 SNP was also negatively associated with logSBP levels. Debette et Visvikis-Siest et al. previously showed a positive relationship between the allele and increased pulse pressure in a healthy population [10]; this could potentially be attributed to the relationship between lower levels of SBP, which would subsequently signify greater values of pulse pressure. On the contrary, the A allele of the rs7043199 variant, which was previously negatively associated with VEGF-A [10,11], was hereby linked with higher levels of logSBP and logDBP. Although not as statistically strong (*p*-value = 0.004), this observed effect could possibly be attributed to the yet-to-be-fully elucidated pleiotropic influence of the variant, the role of which has been previously investigated for overall risk for other disorders related to cardiometabolic profile, namely ischemic heart disease [27] and osteoporosis [28].

To the best of our knowledge, VEGF-A has not been extensively and exclusively examined in adolescents, and the present constitutes the first attempt to construct a uGRS for teenagers using VEGF-A-associated variants. The present 9-SNP uGRS was linked to higher levels of logSBP (Model 1: β = 0.002, *p*-value = 0.03, Model 2: β = 0.019, *p*-value = 0.047, Model 3: β = 0.002, *p*-value = 0.037) and individuals with high GRS presented greater values compared to the ones with low GRS (*p*-value = 0.027), showing that increased genetic predisposition to higher levels of VEGF-A is associated with higher blood pressure in adolescents. This finding is aligned with the well-known relationship between VEGF-A and hypertension, as the current literature has shown that the inhibition of VEGF-A receptors signifies higher levels of circulating VEGF-A, which have, in turn, been associated with a greater risk for hypertension [29,30,31]. In a similar manner and supporting the reciprocal relationship between the VEGF family and hypertension, Zorena et al. showed that adolescents with type 1 diabetes and hypertension displayed greater levels of VEGF compared to healthy individuals or patients with type 1 diabetes but without hypertension [32].

Although this is an overall healthy population with most adolescents presenting normal weight, the accumulating effect of the nine examined SNPs from Choi et al. displayed a statistically significant, positive association with higher logBMI values. In addition to the already underlined positive relationship between VEGF-B and VEGF-C levels and obesity presence [33,34], the current literature further highlights the role of VEGF-A in obesity control [2,35,36]. In the presence of obesity and fat cell proliferation, VEGF-A expression increases as it participates in angiogenesis, cell differentiation and thermogenesis in the white and brown adipose tissues. In this context, VEGF-A contributes to the subsequent increase in energy expenditure and attempts to suppress further diet-induced increase and ameliorate insulin resistance in a compensatory effect [2,35,36]. However, as the increase in adipocytes progresses, VEGF-A is produced more, and angiogenesis is further promoted in the white adipose tissue, thus allowing for further obesity establishment. This cascade of events creates a reciprocal circle where obesity presence induces VEGF-A expression and vice versa. For that reason, the effect of VEGF-A on increased weight can be described as reciprocal and context-dependent, being mainly influenced by the potential pre-existence of increased body weight [1,35]. Hereby, the positive association between the uGRS and logBMI was steadily maintained after adjustments for all three models of confounding factors (Model 1: β = 0.0044, *p*-value = 0.003, Model 2: β = 0.0043, *p*-value = 0.005, Model 3: β = 0.004, *p*-value = 0.009) and adolescents with high versus low genetic risk also presented higher values of logBMI, suggesting an aggravating effect in BMI as a genetic risk for higher VEGF-A increases. In a similar context to the present, Novikova et al. showed that compared to individuals of normal weight, adolescents with obesity presented a 12-fold increase in corresponding VEGF-A levels [37]. To boot, Loebig et al. showed a similar positive association in healthy young men (aged 18–30 years old) under normal blood sugar conditions, where higher levels of VEGF-A were consistently associated with increased weight [38]. VEGF-A was also related to abdominal obesity in a sample of young individuals, as demonstrated by Guzman-Guzman et al. when investigating relations with parameters of the metabolic syndrome [39]. Our present findings show that increased predisposition to higher levels of VEGF-A is related to higher BMI; however, according to the aforementioned, it should be noted that the reciprocity of the relationship remains significant, as increased VEGF-A levels can generally be observed due to increased BMI, thus potentially aggravating the positive predisposing genetic effect.

Another significant relation was observed between the uGRS and lower levels of logHDL (Model 1: β = 0.005, *p*-value = 0.032). Although this association was not maintained after correction for multiple confounding factors, when looking at individuals with higher versus lower genetic risk for increased VEGF-A, the former did present lower values of logHDL. When looking into potential associations between VEGF-A variants and HDL, both Debette et Visvikis-Siest and Stathopoulou et al. showed that the negatively associated with VEGF-A A allele of rs6921438 SNP was related to lower HDL levels in healthy populations [10,12]. The present finding denoting a positive association between increased VEGF-A and lower HDL levels can, thus, potentially be explained by the general overview of the role of elevated VEGF-A in worse lipidemic profile, rather than the direct effect of VEGF-A on HDL per se [40].

Furthermore, taking the biomarker’s role in metabolism into account [2,6,7], we further attempted to unravel the meaning of the interplay between genetic predisposition for higher VEGF-A levels and multiple cardiometabolic indices by examining the potentially modifying role of dietary habits. In our sample, the interaction between the uGRS and the consumption of the “Western Breakfast” was associated with higher levels of logDBP (Model 1: β = 0.0060, *p*-value = 4.28 × 10^−5^, Model 2: β = 0.00568, *p*-value = 0.000239). This finding can be explained by the fact that the “Western Breakfast” pattern consists of food groups with high-fat content, namely cheese, dairy and processed meat [19], which have already been shown to associate with increased blood pressure in the literature [41]. Hojhabrimanesh et al. showed similar significant associations between a “Western” dietary pattern and overall and systolic blood pressure in Iranian adolescents, as well as a positive but not statistically significant association for diastolic pressure [42]. Although the pattern was not unilaterally associated with blood pressure measurements in our team’s previous analyses [19], and an increased predisposition to higher VEGF-A appears to bring its aggravating effect to the forefront and vice versa. This could be partly attributed to the positive effect of the Western diet and red meat-derived protein, which has been previously shown to elevate VEGF-A expression among patients with breast cancer [43].

Furthermore, although the 9-SNP uGRS was not alone associated with glucose in our sample, it did present a nominally significant interaction with the protein-rich “Eggs and Fibers” dietary pattern (consisting of non-refined cereals, vegetables and eggs) in increasing logGlucose levels (Model 2: β = 0.00883, *p*-value = 0.0132). The involvement of VEGF-A in glucose homeostasis is well-known [8], as low levels of the biomarker are linked to insulin resistance, while its overexpression is associated with impaired insulin production and increased glucose levels [2,8]. Consequently, research in adolescent cohorts to date mainly surrounds diabetic individuals or related complications [30,44] and has yet to yield significant results in healthy populations. Although fiber intake is generally regarded as having protective effects in the production of inflammatory biomarkers [45], the present finding could possibly refer to the reciprocal effect of dietary carbohydrate and protein intake on aggravating the genetic risk for VEGF-A levels and subsequent influence the elevated glucose levels.

Moreover, similar gene–diet interactions have also been explored in individuals with metabolic syndrome in studies examining target SNPs for VEGF-A rather than using a holistic genetic risk score approach. Ghazizadeh et al. showed that individuals with the AA genotype for the rs10738760 variant, which was also included in the present uGRS, and higher adherence to foods with increased sugar and saturated fatty acids, among others, presented a greater risk for metabolic syndrome [16]. It was further demonstrated that the presence of the same A allele can significantly interact with even favorable dietary components (e.g., PUFAs) in ultimately elevating the risk for worse glycemic and lipidemic profile and, thus, metabolic syndrome [16]. Taking it one step further, Chedid et al. showed a significant association between BMI and the rs10738760 polymorphism in decreasing iron levels, an effect shown to be more prominent in individuals with obesity [18]. Finally, a different relation concerned the observed associations between the presence of the 9-SNP-uGRS rs6921438 and rs6993770 included SNPs and micronutrient contents, namely high manganese, low zinc, and low iron intakes in patients with metabolic syndrome [46,47,48].

The strengths of the present study concern its hypothesis of investigating demonstrated effects of known VEGF-A variants on the cardiometabolic profile of healthy adolescents for the first time. Various associations presented hereby underline the effect of the SNPs in this age group and further highlight the complementary and modifying effect of diet in this vulnerable and crucial for future development life stage. The limitations of the study are summarized as follows: (i) the limited but substantial number of participants compared to larger cohorts examining VEGF-A-related variants; (ii) the overall health status of the population used, which might not have promoted the identification of distinct associations with cardiometabolic risk factors, as for example in the case of patients with obesity or disrupted glucose metabolism and; (iii) the restricted variance of the populations’ habits explained by the previously extracted dietary patterns (46.69%) [19].

## 5. Conclusions

The results from the present study suggest that well-identified VEGF-A-related variants in adults affect the parameters of adolescent cardiometabolic profiles. Our findings highlight the complexity of the mechanisms in which VEGF-A-related variants affect cardiometabolic risk factors both directly but also potentially through pleiotropic effects. Assessment of the role of diet showed that interaction between genetic makeup and dietary habits could significantly influence the variation of glycemic and blood pressure indices in this age group. In this spectrum, our findings promote the enhancement of our understanding of VEGF-A influence and its individual interaction with dietary aspects. We hereby lay the ground for future GWAS studies to be held that include larger adolescent sample sizes, allowing for the establishment of corresponding effect sizes and the subsequent construction of weighted GRSs for VEGF-A in teenagers. The latter would broaden our abilities in evaluating this reciprocal relationship and even allow for the use of the risk scores as tools of individual and clinical utility in assessing the risk for adolescent cardiometabolic disorders.

## Figures and Tables

**Table 1:** List of the VEGF-A-related single-nucleotide polymorphisms (SNPs) (*n* = 11) investigated for cardiometabolic associations in the TEENAGE cohort.

Consortial Summary Statistics								TEENAGE Cohort	
SNP	Gene	Chr	Position	Alleles	MAF	Effect Allele	Direction of Effect for VEGF	EAF	Ref.
rs114694170	MEF2C, MEF2C-AS1	5	5:88884379	T/C	0.02 (C)	T	Negative (beta = −0.15)	0.96	[6]
rs6921438	SCIRT, LOC100132354	6	6:43957870	G/A/C	0.44 (A)	A	Negative (beta = −0.72)	0.39	[6,7]
rs1740073	LINC02537, SCIRT, C6orf223	6	6:43979661	T/A/C	0.20 (T)	T	Positive (beta = 0.09)	0.35	[6]
rs4416670	SCIRT	6	6:43982716	T/A/C	0.47 (C)	C	Negative (beta = −0.13)	0.44	[7]
rs6993770	ZFPM2-AS1,ZFPM2	8	8:105569300	A/T	0.36 (T)	T	Negative (beta = 0.17)	0.31	[6,7]
rs7043199	VLDLR-AS1	9	9:2621145	T/A	0.11 (A)	A	Negative (beta = −0.10)	0.19	[6]
rs10738760	VLDLR, KCNV2	9	9:2691186	A/G	0.41 (G)	G	Negative (beta = −0.28)	0.46	[7]
rs2375981	VLDLR, KCNV2	9	9:2692583	C/A/G/T	0.41 (G)	C	Positive (beta = 0.21)	0.44	[6]
rs74506613/proxy rs10761741 used	JMJD1C	10	10:63306426	G/T	0.37 (T)	T	Positive (beta = 0.08)	0.47	[6]
rs4782371	ZFPM1	16	16:88502423	T/A/C/G	0.41 (G)	T	Negative (beta = −0.07)	0.36	[6]
rs2639990	ZADH2	18	18:75203596	T/C	0.10 (C)	T	Positive (beta = 0.11)	0.10	[6]

SNP: Single-Nucleotide Polymorphism, Chr: Chromosome, bp: base pairs, MAF: Minor Allele Frequency (as shown in GWAS Catalog), Ref.: Reference.

**Table 2:** Descriptive characteristics of the TEENAGE Study.

	All		Boys		Girls		
	*n*	Median (IQR)	*n*	Median (IQR)	*n*	Median (IQR)	*p*-Value *
Age (years)	766	13.30 (1.31)	349	13.36 (1.38)	417	13.26 (1.25)	<0.001
BMI (kg/m^2^)	766	20.88 (4.38)	349	20.85 (4.45)	417	20.93 (4.37)	0.517
Triglycerides (mg/dL)	611	56.00 (24)	283	55.00 (25)	328	57.00 (24)	0.090
Total Cholesterol (mg/dL)	611	157.00 (33)	283	156.49 (25.18) **	328	157.50 (31)	0.210
SBP (mmHg)	743	119.00 (16)	335	120.67 (11.93) **	408	118.00 (15)	0.001
DBP (mmHg)	743	70.00 (12)	335	71.00 (12)	408	70.00 (12)	0.825
PP	743	47.00 (13)	335	49.23 (10.61) **	408	46 (12)	<0.001
LDL (mg/dL)	611	54.00 (16)	283	90.57 (21.78) **	328	88.40 (26)	0.651
HDL (mg/dL)	611	89.20 (27)	283	53.00 (16)	328	56.00 (17)	0.001
Glucose (mg/dL),	611	80.00 (12)	283	81.00 (11)	328	79.00 (12)	<0.05
CRP (mg/dL)	540	0.30 (1)	254	0.45 (1)	286	0.20 (0)	<0.001

BMI: Body Mass Index, SBP: Systolic Blood Pressure, DBP: Diastolic Blood Pressure, PP: Pulse Pressure, HDL: High-density lipoprotein cholesterol, LDL: Low-density lipoprotein cholesterol, CRP: C-reactive protein. * Hypothesis testing took place via use of the Mann–Whitney test. ** The variable summary statistics are shown as mean ± standard deviation (SD).

**Table 3:** Associations between the 11 VEGF-A-related SNPs and cardiometabolic indices in the TEENAGE cohort.

	Model 1		Model 2		Model 3	
	Beta	*p*-Value	Beta	*p*-Value	Beta	*p*-Value
LogBMI						
rs114694170	0.01009	0.3424	0.01317	0.2385	0.01239	0.2707
rs6921438	−0.00631	0.1131	−0.0053	0.2038	−0.00475	0.2564
rs1740073	0.005531	0.1785	0.003664	0.3826	0.002784	0.5088
rs4416670	−0.00698	0.06125	−0.00389	0.3099	−0.00363	0.3452
rs6993770	−0.00649	0.1252	−0.00866	0.04606	−0.00858	0.0483
rs7043199	−0.01265	0.01352	−0.01202	0.02304	−0.01185	0.02551
rs10738760	0.003147	0.4208	0.002341	0.5588	0.00203	0.6125
rs2375981	0.003426	0.3883	0.002837	0.4846	0.002472	0.5432
rs10761741	0.003055	0.4467	0.003455	0.3978	0.003062	0.4544
rs4782371	0.00442	0.2833	0.003158	0.4576	0.002953	0.4892
rs2639990	−0.00297	0.6463	−0.00232	0.7241	−0.0021	0.7516
logTriglycerides						
rs114694170	0.008907	0.7274	0.02828	0.2978	0.029	0.292
rs6921438	0.001028	0.9184	0.01319	0.2007	0.01328	0.2003
rs1740073	0.006261	0.5473	0.002573	0.8058	0.00253	0.8107
rs4416670	1.83 × 10^−5^	0.9984	0.00513	0.5827	0.004898	0.6018
rs6993770	0.006058	0.5595	−0.00307	0.7726	−0.00332	0.7567
rs7043199	−0.01681	0.1822	−0.01787	0.1588	−0.01938	0.1304
rs10738760	−0.02382	0.01482	−0.0201	0.04157	−0.0201	0.04306
rs2375981	−0.01995	0.04558	−0.01675	0.09515	−0.01696	0.09375
rs10761741	0.004158	0.6738	−0.00254	0.7989	−0.00198	0.844
rs4782371	−0.00071	0.9448	0.00189	0.8571	0.001944	0.8546
rs2639990	−0.01428	0.3776	−0.01309	0.4196	−0.0138	0.4033
logCholesterol						
rs114694170	−0.00314	0.7859	−0.00783	0.5438	−0.00896	0.4916
rs6921438	−0.00051	0.9111	0.000254	0.9586	−9.61 × 10^−5^	0.9844
rs1740073	0.000767	0.8706	0.000225	0.9639	−0.00033	0.947
rs4416670	0.001849	0.6564	0.004052	0.3602	0.004303	0.3322
rs6993770	0.0042	0.3709	0.002885	0.567	0.002729	0.5901
rs7043199	−0.00066	0.908	−9.11 × 10^−5^	0.9879	−0.00107	0.8596
rs10738760	−0.00256	0.5642	−0.00355	0.4489	−0.00351	0.4558
rs2375981	−0.00357	0.4299	−0.00446	0.3497	−0.00424	0.3768
rs10761741	−0.00642	0.1503	−0.00856	0.0695	−0.0087	0.06685
rs4782371	0.003328	0.4736	0.001601	0.7478	0.002173	0.6649
rs2639990	−0.00337	0.645	−0.00521	0.4986	−0.00315	0.6864
logSBP						
rs114694170	0.004856	0.4602	0.01095	0.1322	0.01002	0.1704
rs6921438	−0.00528	0.03273	−0.00571	0.03214	−0.00614	0.02126
rs1740073	0.006211	0.01456	0.007036	0.008435	0.007113	0.007929
rs4416670	−0.00707	0.002172	−0.00744	0.002407	−0.00716	0.003524
rs6993770	−0.005	0.05437	−0.00489	0.07711	−0.005	0.07093
rs7043199	0.007357	0.02104	0.009594	0.004338	0.009446	0.005093
rs10738760	−0.00105	0.6643	−0.00018	0.9445	−0.0002	0.9368
rs2375981	−0.00048	0.8464	0.000475	0.8549	0.000676	0.7948
rs10761741	0.004394	0.07559	0.003574	0.1711	0.003634	0.1643
rs4782371	−0.0017	0.5082	−0.00148	0.5885	−0.00099	0.7192
rs2639990	−0.00027	0.9467	−0.00181	0.6667	−0.00112	0.7913
logDBP						
rs114694170	−0.00538	0.5747	−0.00023	0.9829	−0.00073	0.945
rs6921438	−0.00617	0.08685	−0.00804	0.03627	−0.00845	0.0283
rs1740073	0.005599	0.1311	0.006755	0.07975	0.006983	0.07167
rs4416670	−0.00556	0.09872	−0.00686	0.05272	−0.00661	0.06318
rs6993770	−0.00621	0.101	−0.0043	0.281	−0.00443	0.2685
rs7043199	0.01191	0.01033	0.01359	0.005051	0.0138	0.004611
rs10738760	6.32 × 10^−6^	0.9986	0.001639	0.6575	0.001642	0.6579
rs2375981	−0.00022	0.9508	0.001781	0.6339	0.002048	0.5851
rs10761741	0.005385	0.135	0.006435	0.08701	0.006501	0.0848
rs4782371	0.000505	0.8928	0.002055	0.6027	0.002789	0.4824
rs2639990	0.004213	0.4671	0.003025	0.6163	0.003598	0.5553
logPP						
rs114694170	0.02169	0.1799	0.03011	0.0877	0.02892	0.1044
rs6921438	−0.00429	0.4814	−0.00136	0.8342	−0.00166	0.7989
rs1740073	0.008354	0.1826	0.008206	0.2063	0.007979	0.223
rs4416670	−0.01232	0.03026	−0.01075	0.07144	−0.0104	0.08316
rs6993770	−0.0003	0.9623	−0.00313	0.6417	−0.0031	0.6466
rs7043199	−0.00119	0.8798	0.002393	0.77	0.001466	0.859
rs10738760	−0.0021	0.7244	−0.00156	0.8026	−0.00142	0.8201
rs2375981	−0.00033	0.9559	−0.00017	0.9786	9.90 × 10^−5^	0.9875
rs10761741	0.005041	0.4081	0.000931	0.8832	0.000839	0.8954
rs4782371	−0.00663	0.2943	−0.00846	0.2027	−0.00844	0.2076
rs2639990	−0.00571	0.5596	−0.00865	0.3943	−0.00733	0.4765
logGlucose						
rs114694170	0.01915	0.4259	0.01844	0.488	0.01499	0.5762
rs6921438	−0.00684	0.4689	−0.01078	0.2855	−0.01227	0.2245
rs1740073	0.00942	0.3361	0.007099	0.4879	0.006708	0.5143
rs4416670	0.000832	0.9235	0.000346	0.9698	0.000223	0.9806
rs6993770	−0.01043	0.2856	−0.00569	0.5839	−0.00679	0.5148
rs7043199	0.008424	0.4782	0.008428	0.4973	0.006293	0.6144
rs10738760	0.006866	0.457	0.003822	0.6927	0.002642	0.7852
rs2375981	0.007188	0.445	0.004344	0.6588	0.003512	0.722
rs10761741	0.003465	0.7095	0.004664	0.6322	0.006317	0.5187
rs4782371	−0.01497	0.1213	−0.00968	0.3456	−0.00954	0.3557
rs2639990	−0.00127	0.9336	−0.0042	0.7913	−0.00359	0.8233
logLDL						
rs114694170	−0.0082	0.6443	−0.02002	0.3046	−0.02187	0.2661
rs6921438	−0.00502	0.4711	−0.00418	0.573	−0.00419	0.5718
rs1740073	0.000988	0.8914	−0.00091	0.9035	−0.0022	0.7704
rs4416670	0.001987	0.7558	0.006226	0.3529	0.006893	0.3039
rs6993770	−0.00281	0.6968	−0.00581	0.4461	−0.00551	0.4718
rs7043199	0.006725	0.4431	0.006013	0.5094	0.005337	0.5605
rs10738760	−0.01029	0.1306	−0.01186	0.09438	−0.01145	0.1071
rs2375981	−0.01274	0.06626	−0.01425	0.04787	−0.01372	0.05769
rs10761741	−0.00519	0.4493	−0.00794	0.2667	−0.0091	0.2047
rs4782371	0.01135	0.1115	0.007783	0.3015	0.008257	0.2758
rs2639990	−0.00388	0.7136	−0.00713	0.517	−0.00744	0.5042
logHDL						
rs114694170	0.001151	0.9449	−0.00031	0.9867	−0.00111	0.9524
rs6921438	0.002231	0.7332	0.00056	0.9363	−0.00014	0.9837
rs1740073	0.002099	0.7572	0.005597	0.4303	0.00606	0.3951
rs4416670	0.002402	0.6887	0.000127	0.984	−0.00021	0.9737
rs6993770	0.01151	0.08893	0.0148	0.03953	0.01427	0.04781
rs7043199	−0.00711	0.3875	−0.00429	0.6186	−0.00585	0.4992
rs10738760	0.01409	0.02729	0.01249	0.06206	0.01223	0.06815
rs2375981	0.01261	0.05275	0.01139	0.09454	0.01129	0.09822
rs10761741	−0.01029	0.1098	−0.01098	0.1037	−0.00975	0.15
rs4782371	−0.00762	0.2552	−0.0072	0.3117	−0.0068	0.3417
rs2639990	−0.00388	0.7136	−0.00713	0.517	−0.00744	0.5042
logCRP						
rs114694170	−0.0379	0.6541	−0.04237	0.6554	−0.03521	0.711
rs6921438	−0.0418	0.1947	−0.04414	0.2017	−0.04039	0.241
rs1740073	−0.00433	0.8972	−0.0181	0.606	−0.02466	0.482
rs4416670	−0.0194	0.511	−0.01528	0.6242	−0.0162	0.6012
rs6993770	−0.01718	0.6107	−0.00339	0.9251	−0.0048	0.8941
rs7043199	0.02666	0.5029	0.003378	0.9353	0.000455	0.9913
rs10738760	0.02319	0.4658	0.02242	0.5016	0.02371	0.4762
rs2375981	0.02867	0.3747	0.02603	0.441	0.02572	0.4462
rs10761741	0.0237	0.4588	0.01415	0.6735	0.01207	0.7179
rs4782371	−0.04092	0.2165	−0.03658	0.3002	−0.03689	0.2958
rs2639990	−0.05523	0.2803	−0.05647	0.2884	−0.05193	0.3325

Model 1: Adjusted for age and sex, Model 2: Adjusted for age, sex and exercise, Model 3: Adjusted for age, sex, exercise and dietary patterns. BMI: Body Mass Index, SBP: Systolic Blood Pressure, DBP: Diastolic Blood Pressure, PP: Pulse Pressure, LDL: Low-density cholesterol, HDL: High-density cholesterol, CRP: C-reactive protein.

**Table 4:** Associations between the 9-SNP uGRS and selected cardiometabolic indices in the TEENAGE cohort.

	Model 1			Model 2			Model 3		
	Estimate	SE	*p*-Value	Estimate	SE	*p*-Value	Estimate	SE	*p*-Value
logBMI									
9-SNP uGRS for VEGF-A	0.004445	0.001494	0.00305	0.004349	0.001553	0.005277	0.0040937	0.0015678	0.009281
logTriglycerides									
9-SNP uGRS for VEGF-A	0.005892	0.003854	0.127	0.004260	0.003915	0.2771	0.004650	0.003994	0.2450
logCholesterol									
9-SNP uGRS for VEGF-A	−0.0001979	0.0017479	0.90992	−0.000716	0.001859	0.70024	−0.0007685	0.0018917	0.68474
logSBP									
9-SNP uGRS for VEGF-A	0.002006	0.000924	0.0303	0.0019840	0.0009974	0.047203	0.0020983	0.0010045	0.037205
logDBP									
9-SNP uGRS for VEGF-A	0.001891	0.001351	0.161963	0.002211	0.001441	0.12569	0.002365	0.001455	0.10458
LogPP									
9-SNP uGRS for VEGF-A	0.002425	0.002268	0.2854	0.001599	0.002413	0.50779	0.0015523	0.0024439	0.52558
LogGlucose									
9-SNP uGRS for VEGF-A	0.0009057	0.0036448	0.804	0.001952	0.003840	0.611	0.0028415	0.0038989	0.4665
logLDL									
9-SNP uGRS for VEGF-A	0.003038	0.002688	0.2589	0.002300	0.002818	0.4148	0.001733	0.002863	0.5454
LogHDL									
9-SNP uGRS for VEGF-A	−0.005336	0.002493	0.03279	−0.004999	0.002631	0.05812	−0.004455	0.002673	0.09630
LogCRP									
9-SNP uGRS for VEGF-A	0.001437	0.012397	0.90778	−0.0001663	0.0131008	0.98988	−0.001631	0.013250	0.90207

Model 1: Adjusted for age and sex, Model 2: Adjusted for age, sex and exercise, Model 3: Adjusted for age, sex, exercise and dietary patterns. BMI: Body Mass Index; SBP: Systolic Blood Pressure; DBP: Diastolic Blood Pressure; PP: Pulse Pressure; LDL: Low-density cholesterol; HDL: High-density cholesterol; CRP: C-reactive protein; SE: Standard Error.

**Table 5:** Associations between the 9-SNP uGRS for VEGF-A and dietary patterns in the TEENAGE cohort.

	Model 1 *			Model 2 *		
	Estimate	SE	*p*-Value	Estimate	SE	*p*-Value
logBMI						
uGRS*Western Breakfast	0.0006259	0.0016544	0.70532	0.0009623	0.0016699	0.564684
uGRS*Legumes and Good Fat	0.0004362	0.0014115	0.75742	−0.0002951	0.0015027	0.844375
uGRS*Homemade Meal	−0.001836	0.001302	0.15906	−0.001894	0.001326	0.153652
uGRS*Chicken and Sugars	−0.001955	0.001442	0.17566	−0.001508	0.001577	0.339236
uGRS*Eggs and Fibers	−0.000687	0.001204	0.56840	0.0004325	0.0014616	0.767393
logTriglycerides						
uGRS*Western Breakfast	−0.003976	0.004121	0.335	−0.003394	0.004147	0.4135
uGRS*Legumes and Good Fat	−0.003084	0.003643	0.398	−0.002993	0.003701	0.4192
uGRS*Homemade Meal	−0.0003673	0.0031521	0.907	−0.0004249	0.0031042	0.8912
uGRS*Chicken and Sugars	−0.000562	0.003527	0.873	0.000446	0.003723	0.9047
uGRS*Eggs and Fibers	0.0004714	0.0029163	0.872	−8.952 × 10^−7^	3.645 × 10^−3^	0.9998
logCholesterol						
uGRS*Western Breakfast	−0.0003120	0.0018673	0.86737	−0.0003595	0.0019652	0.85495
uGRS*Legumes and Good Fat	4.399 × 10^−4^	1.654 × 10^−3^	0.79038	0.0006190	0.0017604	0.72529
uGRS*Homemade Meal	0.0022544	0.0014247	0.11421	0.0024594	0.0014679	0.09455
uGRS*Chicken and Sugars	0.0005882	0.0015997	0.71324	0.0011419	0.0017668	0.51840
uGRS*Eggs and Fibers	−0.0024429	0.0013171	0.064231	−0.0035654	0.0017221	0.0390
logSBP						
uGRS*Western Breakfast	0.0019835	0.0010171	0.05164	0.0021791	0.0010716	0.042500
uGRS*Legumes and Good Fat	0.0009800	0.0008694	0.2601	0.001112	0.000966	0.250296
uGRS*Homemade Meal	−0.0004048	0.0008249	0.6238	−0.0006534	0.0008508	0.442827
uGRS*Chicken and Sugars	0.0003776	0.0008987	0.6745	0.0003459	0.0010081	0.731659
uGRS*Eggs and Fibers	−0.0011855	0.0007341	0.1068	−0.0018073	0.0009354	0.053889
logDBP						
uGRS*Western Breakfast	0.0060753	0.0014736	4.28 × 10^−5^	0.005687	0.001537	0.000239
uGRS*Legumes and Good Fat	0.0009039	0.0012713	0.477344	0.001483	0.001396	0.28856
uGRS*Homemade Meal	−0.0008981	0.0012064	0.45691	−0.001097	0.001229	0.37234
uGRS*Chicken and Sugars	1.822 × 10^−5^	1.316 × 10^−3^	0.988960	0.001229	0.001457	0.39932
uGRS*Eggs and Fibers	0.0001876	0.0010752	0.86156	−0.0009524	0.0013559	0.48273
logPP						
uGRS*Western Breakfast	−0.004375	0.002501	0.08081	−0.003179	0.002602	0.22237
uGRS*Legumes and Good Fat	0.0006745	0.0021355	0.75221	0.0001765	0.0023393	0.93989
uGRS*Homemade Meal	0.0001585	0.0020281	0.93772	−5.986 × 10^−5^	2.067 × 10^−3^	0.97691
uGRS*Chicken and Sugars	0.0006736	0.0022094	0.76055	−0.001662	0.002442	0.49637
uGRS*Eggs and Fibers	−0.003235	0.001801	0.07296	−0.002587	0.002269	0.2548
logGlucose						
uGRS*Western Breakfast	−0.0002371	0.0038992	0.952	−0.0006882	0.0040671	0.866
uGRS*Legumes and Good Fat	−0.004075	0.003441	0.237	−0.002575	0.003628	0.478
uGRS*Homemade Meal	−0.0035228	0.0029773	0.237	−0.003946	0.003039	0.195
uGRS*Chicken and Sugars	0.003550	0.003317	0.285	0.003922	0.003634	0.281
uGRS*Eggs and Fibers	5.869 × 10^−3^	2.745 × 10^−3^	0.0330	0.008830	0.003550	0.0132
logLDL						
uGRS*Western Breakfast	−0.0003845	0.0028733	0.8936	−0.0008217	0.0029791	0.7828
uGRS*Legumes and Good Fat	0.001102	0.002545	0.6652	0.001857	0.002669	0.4870
uGRS*Homemade Meal	0.002229	0.002194	0.3103	0.002617	0.002230	0.2412
uGRS*Chicken and Sugars	0.0024563	0.0024563	0.9468	0.0008795	0.0026757	0.7425
uGRS*Eggs and Fibers	−0.004027	0.002024	0.0472	−0.005950	0.002606	0.0229
logHDL						
uGRS*Western Breakfast	0.0007058	0.0026675	0.79145	0.001002	0.002789	0.71958
uGRS*Legumes and Good Fat	0.0004628	0.0023529	0.84413	−7.341 × 10^−5^	2.485 × 10^−3^	0.97644
uGRS*Homemade Meal	0.003719	0.002032	0.06787	0.003693	0.002080	0.07649
uGRS*Chicken and Sugars	0.001880	0.002275	0.40903	0.002321	0.002496	0.3529
uGRS*Eggs and Fibers	−0.0003372	0.0018861	0.85819	−0.0007087	0.0024472	0.77227
logCRP						
uGRS*Western Breakfast	−0.009797	0.013082	0.45430	−0.0072781	0.0136345	0.59379
uGRS*Legumes and Good Fat	0.002883	0.011393	0.80035	−0.0031947	0.0119986	0.79019
uGRS*Homemade Meal	0.010795	0.009823	0.27239	0.011024	0.010010	0.27144
uGRS*Chicken and Sugars	0.004140	0.010979	0.70632	−0.0006592	0.0120081	0.95625
uGRS*Eggs and Fibers	−0.006220	0.008995	0.48963	0.0010038	0.011644	0.93135

* Model 1: Adjusted for age, sex, uGRS and each dietary pattern, Model 2: Adjusted for age, sex, and exercise. uGRS and each dietary pattern. BMI: Body Mass Index; SBP: Systolic Blood Pressure; DBP: Diastolic Blood Pressure; PP: Pulse Pressure; LDL: Low-density cholesterol; HDL: High-density cholesterol; CRP: C-reactive protein; SE: Standard Error.

## Data Availability

Access to the study data is available upon request due to participants’ privacy and ethical restrictions.
